# Study on intestinal parasitic infections and gut microbiota in cancer patients at a tertiary teaching hospital in Malaysia

**DOI:** 10.1038/s41598-024-59969-6

**Published:** 2024-06-13

**Authors:** Sidi Omar Siti Farah Norasyikeen, Romano Ngui, Ab Rahman Syaza Zafirah, Muhammad Zarul Hanifah Md Zoqratt, Wilhelm Wei Han Eng, Qasim Ayub, Syafinaz Amin Nordin, Vesudian Narcisse Mary Sither Joseph, Sabri Musa, Yvonne Ai Lian Lim

**Affiliations:** 1 Department of Parasitology, Faculty of Medicine, Universiti Malaya, 50603 Kuala Lumpur, Malaysia; 2 Department of Paraclinical Sciences, Faculty of Medicine and Health Sciences, Universiti Malaysia Sarawak, 94300 Kota Samarahan, Malaysia; 3 Department of Paediatrics, Faculty of Medicine, Universiti Malaya, 50603 Kuala Lumpur, Malaysia; 4 Department of Microbiology, Faculty of Medicine & Health Sciences, Universiti Putra Malaysia, 43400 Serdang, Malaysia; 5 Monash University Malaysia Genomics Facility, Monash University Malaysia, 47500 Subang Jaya, Malaysia; 6 Department of Children’s Dentistry and Orthodontics, Faculty of Dentistry, Universiti Malaya, 50603 Kuala Lumpur, Malaysia

**Keywords:** Parasitology, Microbiome

## Abstract

Intestinal parasitic infections (IPIs) can lead to significant morbidity and mortality in cancer patients. While they are unlikely to cause severe disease and are self-limiting in healthy individuals, cancer patients are especially susceptible to opportunistic parasitic infections. The gut microbiota plays a crucial role in various aspects of health, including immune regulation and metabolic processes. Parasites occupy the same environment as bacteria in the gut. Recent research suggests intestinal parasites can disrupt the normal balance of the gut microbiota. However, there is limited understanding of this co-infection dynamic among cancer patients in Malaysia. A study was conducted to determine the prevalence and relationship between intestinal parasites and gut microbiota composition in cancer patients. Stool samples from 134 cancer patients undergoing active treatment or newly diagnosed were collected and examined for the presence of intestinal parasites and gut microbiota composition. The study also involved 17 healthy individuals for comparison and control. Sequencing with 16S RNA at the V3–V4 region was used to determine the gut microbial composition between infected and non-infected cancer patients and healthy control subjects. The overall prevalence of IPIs among cancer patients was found to be 32.8%. Microsporidia spp. Accounted for the highest percentage at 20.1%, followed by *Entamoeba* spp. (3.7%), *Cryptosporidium* spp. (3.0%), *Cyclospora* spp. (2.2%), and *Ascaris lumbricoides* (0.8%). None of the health control subjects tested positive for intestinal parasites. The sequencing data analysis revealed that the gut microbiota diversity and composition were significantly different in cancer patients than in healthy controls (p < 0.001). A significant dissimilarity was observed in the bacterial composition between parasite-infected and non-infected patients based on Bray–Curtis (p = 0.041) and Jaccard (p = 0.021) measurements. Bacteria from the genus *Enterococcus* were enriched in the parasite-infected groups, while *Faecalibacterium prausnitzii* reduced compared to non-infected and control groups. Further analysis between different IPIs and non-infected individuals demonstrated a noteworthy variation in *Entamoeba*-infected (unweighted UniFrac: p = 0.008), *Cryptosporidium*-infected (Bray–Curtis: p = 0.034) and microsporidia-infected (unweighted: p = 0.026; weighted: p = 0.019; Jaccard: p = 0.031) samples. No significant dissimilarity was observed between *Cyclospora*-infected groups and non-infected groups. Specifically, patients infected with *Cryptosporidium* and *Entamoeba* showed increased obligate anaerobic bacteria. *Clostridiales* were enriched with *Entamoeba* infections, whereas those from *Coriobacteriales* decreased. *Bacteroidales* and *Clostridium* were found in higher abundance in the gut microbiota with *Cryptosporidium* infection, while Bacillales decreased. Additionally, bacteria from the genus *Enterococcus* were enriched in microsporidia-infected patients. In contrast, bacteria from the *Clostridiales* order, *Faecalibacterium*, *Parabacteroides*, *Collinsella*, *Ruminococcus*, and *Sporosarcina* decreased compared to the non-infected groups. These findings underscore the importance of understanding and managing the interactions between intestinal parasites and gut microbiota for improved outcomes in cancer patients.

## Introduction

Intestinal parasitic infections (IPIs) are caused by parasites that inhabit the intestines, such as protozoa and helminths. They affect about 3.5 billion people globally, especially those in impoverished areas of developing countries^1^. IPIs pose a significant health risk, leading to conditions like anaemia, iron deficiency, stunted growth in children, and various mental and physical health issues. Symptoms include diarrhoea, nausea, abdominal pain, and dysentery, which can result in substantial illness and mortality^2^. Cancer is a severe non-communicable disease affecting people worldwide, with increasing cases, burdening healthcare. Treatments like chemotherapy and radiotherapy can raise the risk of opportunistic infections, including intestinal parasites, in cancer patients. Studies have revealed that immunocompromised individuals are susceptible to chronic diarrhoea caused by these infections^3^.

While cancer patients face similar infection risks, they may struggle to eliminate parasites from their bodies after exposure, leading to severe or disseminated infection. In Malaysia, a study by Menon et al.^4^ found that the overall prevalence of intestinal parasites among children with cancer in Kelantan was 42%, with helminths being the most common infection, followed by protozoa. *Trichuris trichiura* was the most observed parasite, followed by *Ascaris lumbricoides*, *Giardia lamblia*, *Blastocystis hominis*, and hookworm (4). Other epidemiological studies on IPIs among cancer patients in Malaysia reported the prevalence of microsporidia (21.9–68%)^5,6^, *Strongyloides stercoralis* (0.02–0.5%)^7,8^, and *Cryptosporidium* spp. (0.1%)^9^.

Intestinal parasites coexist with gut bacteria, suggesting the gut microbiota’s potential role in influencing IPIs pathophysiology. The gut microbiota, essential for maintaining homeostasis and human health, plays a crucial role in shaping the immune system and cellular metabolism, protecting against opportunistic pathogens, forming new blood vessels, repairing epithelial cell injury, managing energy metabolism, and obtaining essential nutrients^10^. However, the effects of IPIs on the microbial ecology of the human gut are not yet fully understood. Most research has reported associations using animal models^11^, but recent human studies have shown changes in microbial communities in response to intestinal parasites. Depending on the specific type of intestinal parasite in the gut, there may be potentially advantageous or disadvantageous effects on the gut composition^12^. Mejia et al.^13^ found that co-infection with protozoa and helminths reduced microbiota diversity, while helminth infection alone increased diversity. However, research on children in Colombia^14^ revealed that a protozoan species called *Cryptosporidium* spp. did not significantly alter the bacterial microbiota. These findings suggest that the gut microbiota plays a crucial role in the pathophysiology of these infections, potentially leading to inflammation, malabsorption, changes in the microbial community (dysbiosis), and their association with inflammatory bowel disease (IBD), colon cancer (CRC), and Crohn’s disease^15^.

Considering the crucial role of the gut microbiota in human health and defence against infections, it is conceivable that a parasite–microbiota interaction occurs, influencing the course, severity, and symptoms. Understanding the gut microbiota could also aid in infection treatment through dietary management significantly affecting the gut microbiota and using probiotics, prebiotics, anthelmintics, or other future therapeutic approaches. A deep understanding of cancer epidemiology is crucial to identify potential causes and trends among populations affected. This knowledge can help develop appropriate healthcare intervention policies for prevention, screening, and diagnosis. The current study aims to determine intestinal parasites’ prevalence and association with gut microbiome diversity among cancer patients. This research is the first in Malaysia to explore the correlation between parasites and gut microbiota in cancer patients. This study is unique because there is currently insufficient research on the impact of intestinal parasites on the gut microbiota of cancer patients, particularly in Malaysia. While several studies have indicated the presence of dysbiosis in cancer patients’ gut microbiota, intestinal parasites’ effects on a dysbiotic gut remain largely unknown. This study aims to explore how different parasites may modify gut microbiota and their impact on the gut composition of cancer patients in Malaysia. Although dysbiosis has been observed in cancer patients, understanding the relationship between parasitic infections, gut microbiota, and cancer is constantly evolving. It is a complex area requiring ongoing research to grasp the underlying mechanisms fully. We anticipate that this study will offer valuable insights and pave the way for future research to address the limitations of this investigation.

## Results

### General characteristics and the prevalence of parasites

A total of 134 stool samples were examined using microscopy to detect intestinal parasites (Supplementary Table S1). The patient ages ranged from 1 to 96 years, with a median age of 25. Most samples (48.5%; 65/134) were from patients aged 1 to 20 years. Male patients accounted for 56% (75/134) of the samples, and Malay patients were the most frequently sampled ethnic group, making up 44.8% (60/134). Among the patients, 47.8% (59/134) did not display any symptoms, 41% (55/134) had diarrhoea, 9% (12/134) experienced more than one gastrointestinal symptom, and 2.2% (3/134) reported nausea. Detailed information on the types of cancer was not available for 81 of the samples. Of the available data, 40.3% (54/134) were uncharacterised solid tumours, and 20.1% (27/134) were uncharacterised leukaemia. Overall, 52.2% (70/134) were categorised as solid tumours, and 47.8% (64/134) were haematological malignancies. Most samples (88.8%; 119/134) were from patients who had undergone cancer treatment.

Of the 134 stool samples, 43 (32.1%) tested positive for IPIs, as indicated in Table 1. Of the 43 positive samples, 27 (20.1%) were positive for microsporidia spp., 5 (3.7%) for *Entamoeba* spp., 4 (3%) for *Cryptosporidium* spp., 4 (2.2%) for *Cyclospora* spp., and 1 (2.2%) for *A. lumbricoides*. Out of the positive samples, three were co-infected with Microsporidia spp. One of these cases (0.8%) showed co-infection of microsporidia spp. with *Entamoeba* spp. and *Cyclospora* spp., another case (0.8%) showed co-infection of microsporidia spp. with *Entamoeba* spp. and *A. lumbricoides*, while the third case (0.8%) showed co-infection of microsporidia spp. with *Cyclospora* spp.

**Table 1:** Overall prevalence of intestinal parasitic infections (IPIs) based on microscopy (N = 134).

Intestinal parasites	n (%)	95% CI
Single infection		
Protozoa		
*Microsporidia* spp.	27 (20.1)	14.2–27.7
*Entamoeba* spp.	5 (3.7)	1.6–8.4
*Cryptosporidium* spp.	4 (3)	1.2–7.4
*Cyclospora* spp.	3 (2.2)	0.8–6.4
Helminth		
*A. lumbricoides*	1 (0.7)	0.1–4.1
Co-infection		
Microsporidia spp. + *Entamoeba* spp. + *Cyclospora* spp.	1 (0.7)	0.1–4.1
Microsporidia spp. + *Entamoeba* spp. + *Cyclospora* spp. + *A. lumbricoides*	1 (0.7)	0.1–4.1
Microsporidia spp. + *Cyclospora* spp.	1 (0.7)	0.1–4.1
Total	43 (32.1)	25.5–41.2

*N* total number of samples, *n* number of positive samples, *95% CI* confidence interval.

### Microbial profile of cancer patients in Malaysia

In this study, out of 43 samples that tested positive for parasites, 33 parasite-infected cancer patients and 20 non-infected cancer patients, along with 17 healthy controls, were included in the microbiota analysis. The remaining 10 positive samples were excluded due to difficulties in DNA extraction from small stool samples. Five samples were excluded after sequence processing and sample filtering to ensure an even number of reads per sample. Additionally, due to differences in characteristics between cancer patient groups and the influence of host phenotypic variables on gut microbiota composition, all symptomatic patients were excluded to investigate the effects of IPIs on cancer microbial profiles at an asymptomatic level.

The final sample size for the study was 51, with 23 parasite-infected cancer patients compared to 11 non-infected cancer patients and 17 healthy controls. The demographic characteristics of the final study participants are provided in Table 2. Most of the parasite-infected samples contained protozoa infections, with microsporidia spp. (n = 7; 30.4%) being predominant, followed by *Entamoeba* spp. (n = 5; 21.7%), *Cryptosporidium* spp. (n = 4; 17.4%), *Cyclospora* spp. (n = 3; 13.0%), and *A. lumbricoides* (n = 1; 4.3%). Some samples were infected with more than one type of parasite (n = 3; 13.0%).

**Table 2:** Demographic characteristics of the study population.

Characteristics	Overall (N = 51)	Cancer (N = 34)		Control (N = 17)
		Parasite positive (N = 23)	Parasite negative (N = 11)	
Age				
Range	1–17	1–17	1–17	7–10
Median (IQR)	8 (4–10)	6 (4–12.5)	4 (2–7)	9 (8–9)
Mean (SD)	7.62 (4.37)	7.91 (5.43)	5.63 (4.88)	8.53 (1.01)
Age groups				
≤ 3	10 (19.6%)	5 (21.7%)	5 (45.5%)	0 (0.0%)
4–6	9 (17.6%)	6 (26.1%)	3 (27.3%)	0 (0.0%)
7–12	24 (47.1%)	6 (26.1%)	1 (9.1%)	17 (100.0%)
13–18	8 (15.7%)	6 (21.6%)	2 (18.2%)	0 (0.0%)
Gender				
Male	28 (54.9%)	14 (60.9%)	7 (63.6%)	7 (41.2)
Female	23 (45.1%)	9 (39.1%)	4 (36.4%)	10 (58.8)
Race				
Malay	41 (80.4%)	18 (78.3%)	9 (81.8%)	14 (82.4%)
Chinese	6 (11.8%)	5 (21.7%)	1 (9.1%)	0 (0.0%)
Indian	1 (2.0%)	1 (4.3%)	1 (9.1%)	0 (0.0%)
Other	3 (5.9%)	0 (0.0%)	0 (0.0%)	3 (17.6%)
Types of cancer				–
Solid tumour				
Medulloblastoma	3 (5.9%)	2 (8.7%)	1 (9.1%)	
Ewing sarcoma	2 (3.9%)	2 (8.7%)	0 (0.0%)	
Osteosarcoma	2 (3.9%)	1 (4.3%)	1 (9.1%)	
Germinoma	2 (2.0%)	1 (4.3%)	1 (9.1%)	
Angiofibroma	1 (2.0%)	1 (4.3%)	0 (0.0%)	
LCH	1 (2.0%)	1 (4.3%)	0 (0.0%)	
Uncharacterised solid tumour	1 (2.0%)	1 (4.3%)	0 (0.0%)	
Blood cancer				
ALL	15 (29.4%)	9 (39.1%)	6 (54.5%)	
AML	4 (7.8%)	4 (17.4%)	0 (0.0%)	
Lymphoma	1 (2.0%)	1 (4.3%)	0 (0.0%)	
JMML	1 (2.0%)	0 (0.0%)	1 (9.1%)	
Uncharacterised leukaemia	1 (2.0%)	0 (0.0%)	1 (9.1%)	
Cancer group				–
Solid tumour	12 (23.5%)	9 (39.1%)	3 (27.3%)	
Haematological malignancies	22 (43.1%)	14 (60.9%)	8 (72.7%)	
Cancer treatment				–
Yes	22 (43.1%)	14 (60.9%)	7 (63.6%)	
No	12 (23.5%)	9 (39.1%)	4 (36.4%)	
Down syndrome				–
No	48 (94.1%)	21 (91.3%)	10 (90.9%)	
Yes	3 (5.9%)	2 (8.7%)	1 (9.1%)	
Single infections				
Protozoa				
*Microsporidia* spp.	7 (13.7%)	7 (20.6%)		
*Entamoeba* spp.	5 (9.8%)	5 (14.7%)		
*Cryptosporidium* spp.	4 (7.8%)	4 (11.8%)	–	–
*Cyclospora* spp.	3 (5.9%)	3 (8.8%)		
Helminth				
*A. lumbricoides*	1 (2.0%)	1 (2.9%)		
Co-infection				
Microsporidia spp. + *Entamoeba* spp. + *Cyclospora* spp.	1 (2.0%)	1 (2.9%)		
Microsporidia spp. + *Entamoeba* spp. + *Cyclospora* spp. + *A. lumbricoides*	1 (2.0%)	1 (2.9%)	–	–
Microsporidia spp. + *Cyclospora* spp.	1 (2.0%)	1 (2.9%)		

*N* total number of samples, *IQR* Interquartile range, *SD* standard deviation, *ALL* acute lymphoblastic leukaemia, *AML* acute myeloid leukaemia, *JMML* juvenile myelomonocytic leukaemia, *LCH* Langerhans cell histiocytosis.

**Figure 1:**
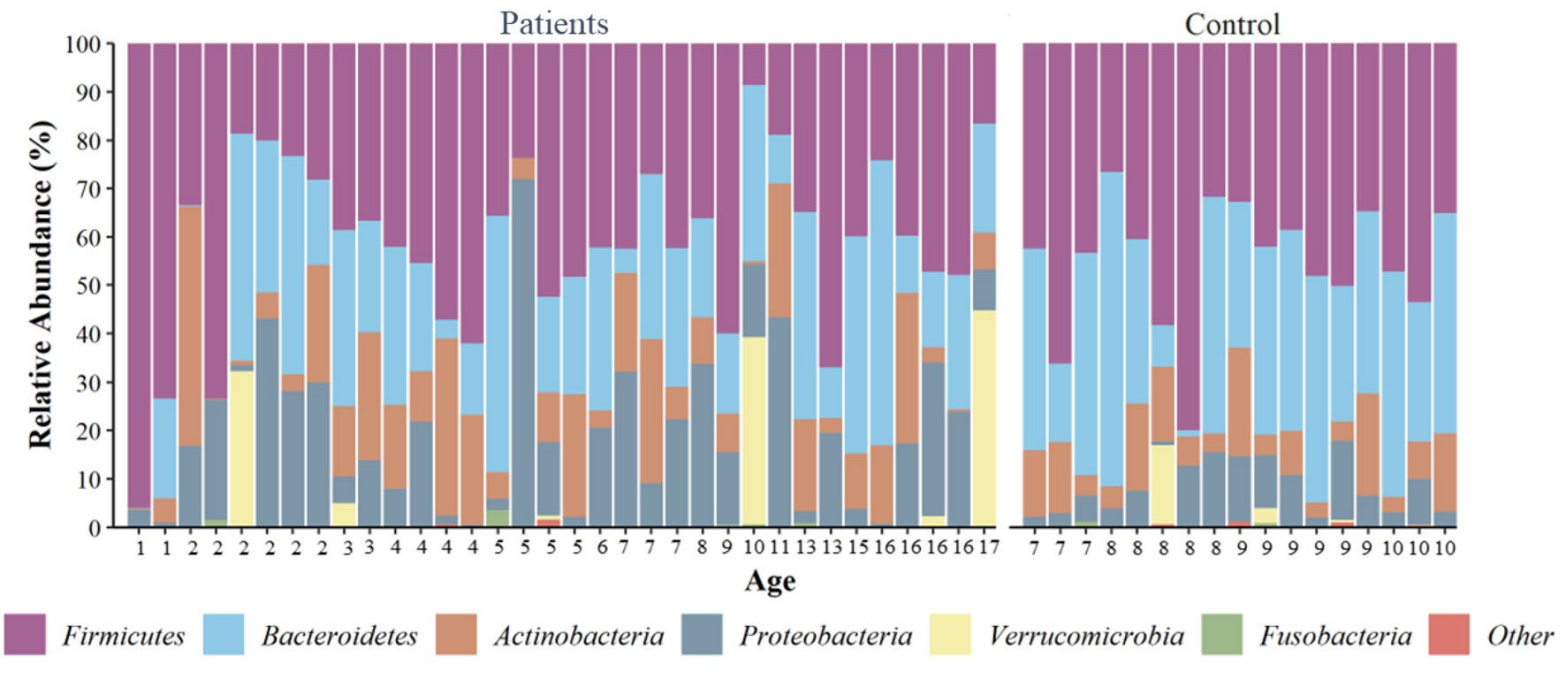
Bacteria phyla compositions among patients with cancer. Age-related stacked bar plots were used to display the distribution of the most abundant phyla in each patient sample. These top 6 phyla (*Firmicutes*, *Bacteroidetes*, *Actinobacteria*, *Proteobacteria*, *Verrucomicrobia* and *Fusobacteria*) made up almost 99% of the abundance, with the remaining taxa being categorised as ‘other’.

Of the 51 included samples, 2,315,116 quality-filtered sequences were obtained, averaging 45,394 ± 31,358 sequences per sample. The sample was rarefied to 5191 reads (minimum sampling depth), and 5746 amplicon sequence variants (ASVs) were obtained. The relative abundance of bacterial phyla in all samples showed that the microbiota composition in every study participant was dominated by the phyla *Firmicutes* (45.4%), followed by *Bacteroidetes* (35.6%), *Proteobacteria* (17.3%), *Actinobacteria* (13.6%), *Verrucomicrobia* (3.63%), and *Fusobacteria* (0.2%) (Fig. 1).

### Association between intestinal parasite infection and gut microbiota

**Figure 2:**
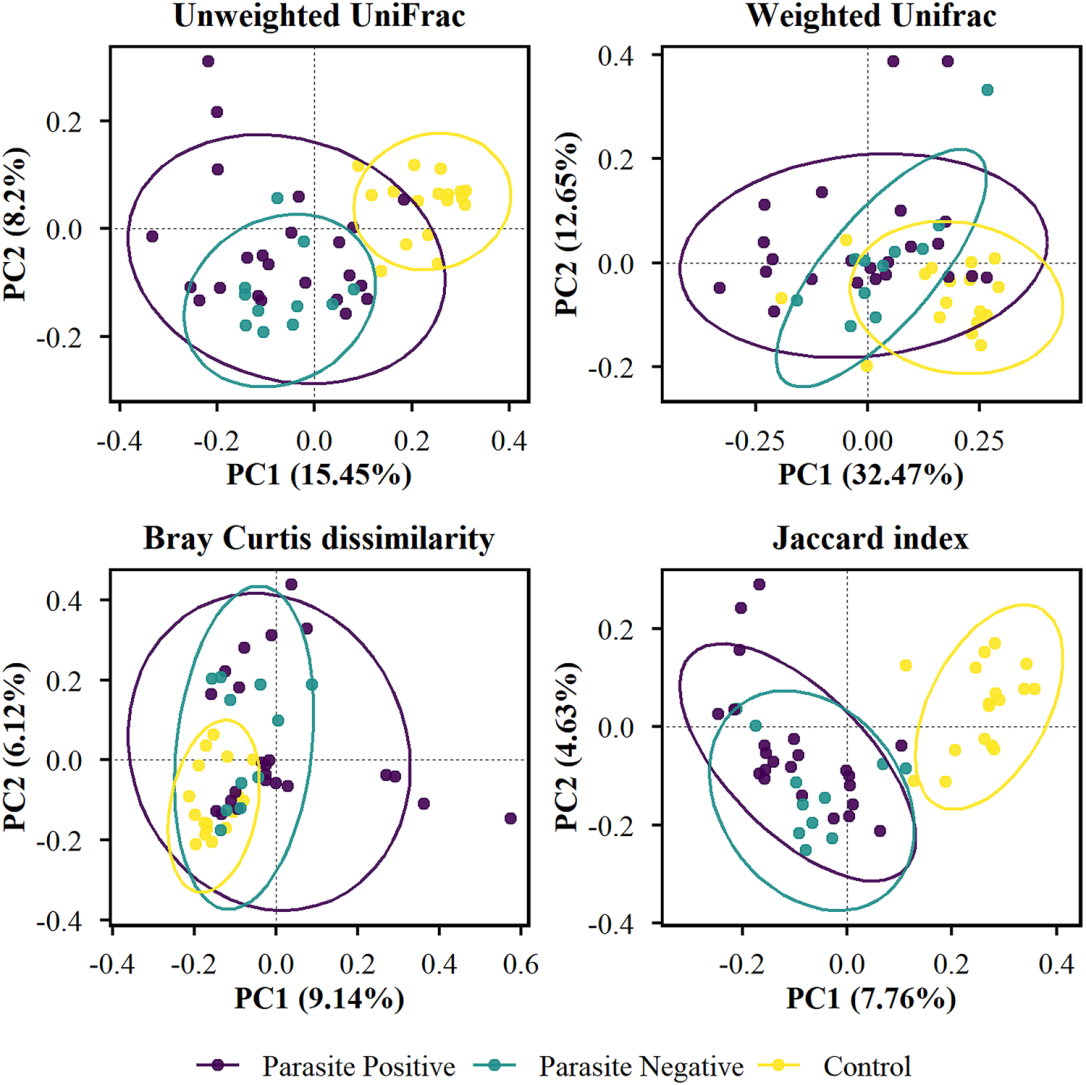
PCoA plot of the microbial communities in parasite-infected and non-infected patients with healthy control as the baseline. The significant separation between parasite-infected and non-infected patients based on Bray–Curtis and Jaccard index distance matrices. Unweighted UniFrac (pseudo-F = 1.31, p = 0.096), weighted UniFrac (pseudo-F = 1.08, p = 0.341), Bray–Curtis: pseudo-F = 1.37, p = 0.041 and Jaccard: pseudo-F = 1.27, p = 0.021).

The PCoA plot of all distance matrices shows parasite-infected and non-infected clusters separate from the control sample (Fig. 2). Moreover, based on Bray–Curtis and Jaccard index distance matrices, a significant dissimilarity was observed in the microbial composition between parasite-infected and non-infected patients. The PERMANOVA supported these findings with 999 permutations (Table 3). Further analysis between different IPIs and non-infected individuals demonstrates a noteworthy variation in *Entamoeba*-infected and *Cryptosporidium*-infected samples, as indicated by unweighted UniFrac (pseudo-F = 1.66; p = 0.008) and Bray–Curtis (pseudo-F = 1.42; p = 0.034) distance matrices, respectively. The structure of microbial communities between microsporidia-infected and non-infected individuals also appears to be dissimilar, as observed in three distance matrices (unweighted; pseudo-F = 1.56, p = 0.026; weighted: pseudo-F = 2.43, p = 0.019; Jaccard: pseudo-F = 1.35, p = 0.031). No significant dissimilarity was observed between *Cyclospora*-infected groups and non-infected groups (Table 3).

**Table 3:** Pairwise PERMANOVA statistics between intestinal parasitic infections and non-infected patients.

Group 1	Group 2	Unweighted UniFrac		Weighted UniFrac		Bray Curtis		Jaccard index	
		pseudo-F	p-value	pseudo-F	p-value	pseudo-F	p-value	pseudo-F	p-value
Parasite positive	Parasite negative	1.31	0.1	1.08	0.34	1.37	0.04	1.27	0.02*
	Healthy control	6.49	0.001*	6.29	0.001*	2.94	0.001*	3.99	0.001*
Parasite negative	Healthy control	6.26	0.001*	3.8	0.002*	2.5	0.001*	3.33	0.001*
Microsporidium spp.	Parasite negative	1.56	0.03*	2.43	0.02*	1.34	0.09	1.35	0.03*
	Healthy control	5.75	0.001*	7.15	0.001*	2.37	0.001*	3.22	0.001*
*Entamoeba* spp.	Parasite negative	1.66	0.01*	0.75	0.64	1.28	0.14	1.16	0.16
	Healthy control	3.08	0.001*	3.31	0.01	2.16	0.001*	2.2	0.001*
*Cryptosporidium* spp.	Parasite negative	1.05	0.35	1.66	0.14	1.42	0.03*	1.07	0.25
	Healthy control	3.05	0.002*	2.13	0.04	1.77	0.003*	2.09	0.001*
*Cyclospora* spp.	Parasite negative	1.29	0.11	0.95	0.48	1.25	0.15	1.12	0.16
	Healthy control	2.74	0.001*	2.47	0.02	1.89	0.002*	1.98	0.001*

Pseudo-F = effect size; Calculated using pairwise PERMANOVA with 999 permutation tests.

*Significant difference *p* ≤ 0.05.

**Figure 3:**
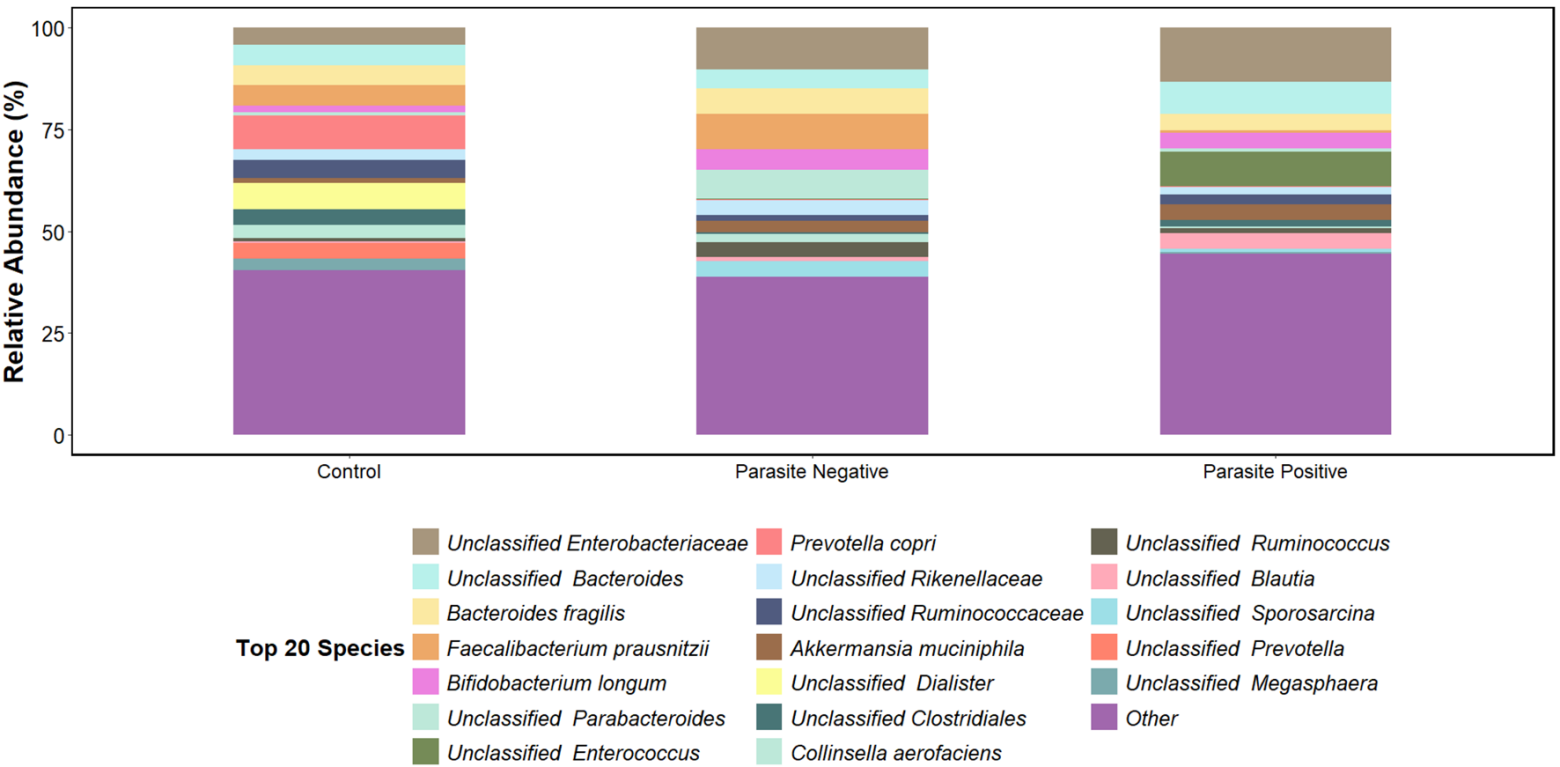
Comparison of microbial composition in parasite-infected, non-infected and healthy control at species level. Age-related stacked bar plots were used to display the distribution of the 20 most abundant phyla in the patient sample group by parasite infection, with the remaining taxa being categorised as ‘other’.

Taxonomic analysis at the species level comparing parasite-infected and non-infected patients with control shows disparity in the prevalence of bacterial species (Fig. 3). Specifically, the relative abundance of unclassified bacteria species within the genus *Enterococcus* was significantly higher in the infected group (8.5%) compared to both the non-infected (0.196%) and control groups (0.001%), with a significant difference of p < 0.001. Conversely, *Faecalibacterium prausnitzii* demonstrated a significantly lower relative abundance in the parasite-infected group (0.576%) when compared to the non-infected (8.65%; p < 0.001) and control group (5.12%; p = 0.03). No significant difference was observed when comparing the relative abundance of *F. prausnitzii* between non-infected patients and the control (p > 0.05). Additionally, the relative abundance of *Prevotella copri* was significantly reduced in both infected (0.0036%; p < 0.001) and non-infected (0.051%; p = 0.01) patients when compared to the control (8.28%).

**Figure 4:**
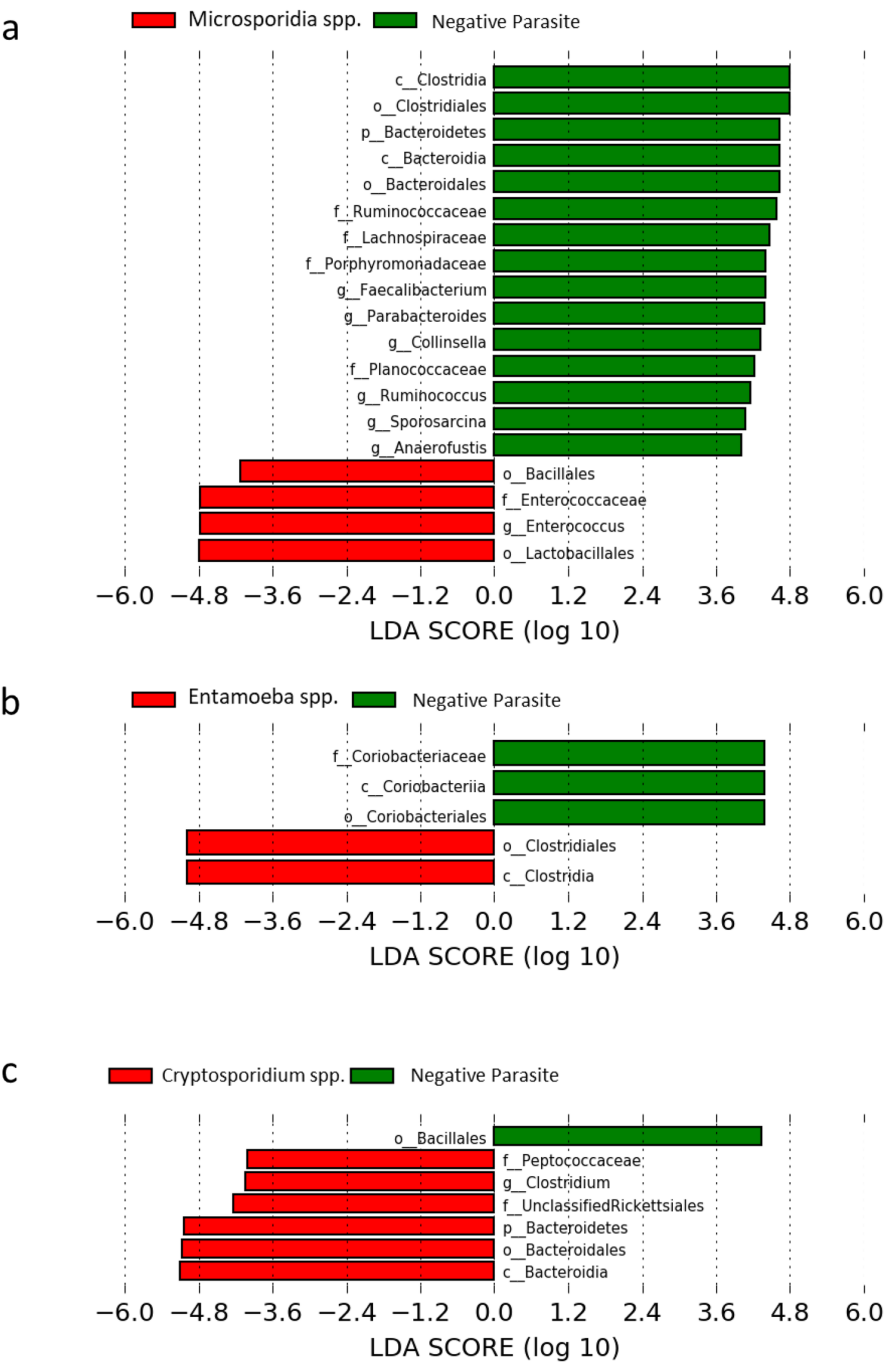
Different abundances of microbial communities between (**a**) *Microsporidium* spp., (**b**) *Entamoeba* spp. and (**c**) *Cryptosporidium* spp. compared to non-infected groups. A cladogram displays the relationship between the significantly distinct taxa at different tiers with the clade as a group of organisms that shares a common ancestor. *Significant difference LDA score ≥ 4.0, p ≤ 0.05.

To identify taxonomic differences associated with intestinal parasites, we used the LEfSe algorithm to compare different types of intestinal parasites significantly different from non-infected samples, as observed in the beta analysis. Based on LEfSe analysis, bacteria from the *Enterococcus* genus and *Bacillales* order were enriched in microsporidia-infected samples, with concurrent decreases in bacteria from the *Clostridiales* order, *Faecalibacterium*, *Parabacteroides*, *Collinsella*, *Ruminococcus*, and *Sporosarcina*. Meanwhile, only minor changes were observed in the gut microbiota based on beta diversity in individuals infected with *Entamoeba* spp. and *Cryptosporidium* spp. Bacteria from *Clostridiales* were enriched with *Entamoeba* infections, whereas those from *Coriobacteriales* decreased. *Bacteroidales* and *Clostridium* were found in higher abundance in the gut microbiota with *Cryptosporidium* infection, while *Bacillales* decreased (Fig. 4).

## Discussion

Recent research has shown changes in gut microbiota diversity and composition in response to intestinal parasites^12–14^. However, most studies have been conducted in animal models, with only a few in humans^12,13^. These changes can have positive or negative effects on overall health, depending on the types of parasites present in the gut. Cancer patients are often susceptible to opportunistic infections, including intestinal parasites. Several studies have shown gut microbiota dysbiosis in cancer patients^16^. However, little is known about the influence of intestinal parasites on gut microbiota among cancer patients in Malaysia. Therefore, this study aims to understand gut microbiota composition among cancer patients with intestinal parasites. Our study revealed significant alpha and beta diversity differences between non-infected cancer patients and healthy controls. These findings support the hypothesis that gut microbiota diversity is reduced in a disease state, and the microbial composition differs from that of healthy individuals^17^. Parasitic infections had little effect on the alpha microbial diversity compared to non-infected patients. However, there was a significant dissimilarity in the gut microbiota composition between parasite-infected and non-infected patients, possibly driven by *Cryptosporidium* spp., *Entamoeba* spp., and microsporidia infections.

The results of this study demonstrated that intestinal parasitic infections (IPIs) can influence gut microbiota, as confirmed by previous studies^12–14^. However, the relationship between intestinal microbiota diversity may depend on which parasite is present in the gut^18^. Notably, studies have also shown that *Giardia* spp. or *Entamoeba* spp. alone can alter microbial communities^12,14^. For example, von Huth et al.^12^ reported that helminth infections largely did not affect microbial diversity, while protozoan infections such as *Entamoeba* spp. and *Giardia* spp. moderately affected the alpha diversity. In a study among asymptomatic children in Argentina, gut microbiota diversity decreased with increased *Giardia* burden or co-infection with *Giardia*-helminth but increased with helminth infection alone^13^. Meanwhile, in Toro-Londono et al.’s^14^ study, *Cryptosporidium* spp. showed no significant alterations to the bacterial microbiota regarding diversity and structure among children in Colombia. *Cryptosporidium* and *Entamoeba* both multiply in the intestinal enterocytes.

Consequently, the impact of these infections on the gut microbiota community could be indirect, as the parasites adhere to and lyse the colonic epithelium. Various studies have shown an increased abundance of bacteria that produce metabolites, representing the microbial response to mucosal or epithelial damage caused by these infections^14^. In this study, we observed a significant enrichment of the taxa *Clostridium* in *Cryptosporidium* infections. Similarly, the Clostridiales order increased in *Entamoeba* infections but decreased in abundance with microsporidia infections. *Clostridium* spp. is the primary commensal bacterial cluster in the gut that synthesises important metabolites such as butyrate, indole propionic acids, and secondary bile acids, which play a crucial role in maintaining intestinal homeostasis^19^. It has been noted that they reduce allergic reactions and inflammation.

Previous studies have reported contradictory findings regarding the abundance of *Clostridium* in helminths^20,21^ and protozoan infections^12,21^. As previously reported, von Huth et al.^12^ observed an increase in the abundance of bacteria from two Clostridium clades (*Clostridium* IV & VIVb) and a decrease in one *Clostridium* clade (*Clostridium* XVIII) based on the types of protozoan infections. In northern India, the abundance of *Clostridiales* decreases in *E. histolytica*-infected patients due to acute or chronic diarrhoea^21^. In mixed infections with *T. trichiura* and *A. lumbricoides*, the abundance of bacteria from the genus *Clostridia* class decreases among children^20^. However, Easton et al.^22^ reported a significant increase in the proportion of *Clostridiales* following albendazole treatment and helminth clearance. These parasites are likely to influence gut physiology indirectly or directly; hence, the number of *Clostridium* spp. has decreased, as seen in cases of intestinal failure and ulcerative colitis.

Additionally, bacteria from the *Bacteroidales* order were also enriched with *Clostridium* in *Cryptosporidium* infections, while the *Bacillales* decreased. Briefly, bacteria from the *Bacteroidales* order include *Bacteroides, Prevotella*, and *Parabacteroides* genera. *Bacteroides* are the predominant genus from the phyla *Bacteroidetes* in the gut microbiota of humans^23^. They can express polysaccharide A, which can stimulate the development of regulatory T cells and the production of cytokines that protect against colitis^24^. These findings may imply that *Cryptosporidium* spp. provides beneficial effects to asymptomatic cancer patients, consistent with previous findings^14,25^. Similarly, bacteria such as *Coriobacteriaceae* and *Lactobacillus* increased in abundance in the gut of asymptomatic mice carriers with *Cryptosporidium* spp.^25^.

A study using an animal model found that antibiotics and diarrhoea may worsen the severity of the disease^26–28^. For example, Lactobacillus bacteria increased in untreated mice infected with *Cryptosporidium* but declined in mice pre-treated with antibiotics such as cloxacillin^27^. Additionally, a study on goats with mild to severe clinical symptoms of cryptosporidiosis, including growth retardation, diarrhoea, hypothermia, and mortality, showed a depletion of bacteria relevant to the synthesis of SCFA^28^. In a study by Carey et al.^26^ among Bangladeshi children, low abundances of *Megasphaera* were associated with diarrheal symptoms before and during *Cryptosporidium* infections compared to subclinical samples^26^.

Two other studies have found a similar relationship between the abundance of *Coriobacteriaceae* and *Entamoeba* infections. Yanagawa et al.^29^ reported high levels of *Coriobacteriaceae*, *Ruminooccaceae*, and *Clostridiaeae*, and a low abundance of *Streptococaceae* in asymptomatic *E. histolytica* infections. On the other hand, von Huth et al.^12^ observed a decrease in *Collinsella* and *Clostridium XVIII,* while *Clostridium IV* increased in individuals infected with *E. histolytica*. These reported differences in findings could be due to geographical variations in gut microbiota. However, both studies confirm that *Entamoeba* infections, similar to *Cryptosporidium* infections, increase the number of beneficial bacteria in their hosts. Furthermore, Verma et al.^21^ observed a significant reduction in the abundance of metabolite-producing bacteria such as *Bacteroides*, *C. coccoides* subgroup, *C. leptum* subgroup, *Lactobacillus*, *Campylobacter*, and *Eubacteroim* and an increase in *Bifidobacterium* while no change in *Ruminococcus* in patients with amoebic dysentery, which worsened the disease severity.

On the other hand, this study also attempts to determine the gut microbiota composition with microsporidia infections. To our knowledge, no prior research has looked at the impact of microsporidian infections on the human gut microbiota. At the same time, reports about the correlations mostly use animal models^30–32^. In this study, we observed the enrichment of opportunistic pathogens such as *Enterococcus* and *Bacillales* in microsporidia infections with a concurrent decrease in abundance of beneficial bacteria such as *Collinsella*, *Parabacteroides*, *Clostridiales* (i.e. *Faecalibacterium*, *Ruminococcus*), and *Sporosarcina*. Based on our findings, microsporidia infections negatively correlate with patient health. Unlike in *Cryptosporidium* and *Entamoeba* infections, this study observed an increased risk for opportunistic infections by *Enterococcus* and *Bacillales* in asymptomatic patients with microsporidian infections while metabolites-producing bacteria depletes.

Based on a previous study, microsporidia infections positively correlated with lactic acid bacteria from the genus *Weissella*^31^. According to Trzebny, except for acid-tolerant species, microsporidians may reduce the gut pH and inhibit most bacteria growth. Our results concur with these observations based on the enrichment of the two taxa (i.e., *Enterococcus* and *Bacillales*). *Enterococcus* spp. and *Bacillales* (particularly *Staphylococcus*) bacteria can survive in an acidic environment^33,34^. Furthermore, microsporidia infections in silkworms have similarly reported enrichment of *Enterococcus* spp. such as *E. faecalis* LX10^32^. Interestingly, Zhang reported that *E. faecalis* benefits silkworms by reducing microsporidia spp. spore germination rate and infection efficiency. Furthermore, *E. faecalis* produces lactic acid, which reduces the gut pH, thus inhibiting silkworm germination and lowering gut injury in silkworms. Therefore, the current research observed that the types of effects seen by intestinal protozoan could be different. While previous studies show *Giardia* and *Entamoeba* are the only protozoan species capable of changing the gut microbiota alone, this study provides evidence that protozoa such as *Cryptosporidium* and microsporidia are capable of affecting the gut microbiota of patients with cancer.

Furthermore, this study shows that both *Cryptosporidium* spp. and *Entamoeba* spp. could have beneficial effects on the human host while asymptomatic but can be negatively correlated when accompanied by antibiotics and diarrhoea. Additionally, this study has observed that microsporidia spp. promotes enrichment of possibly opportunistic pathogenic bacteria with a reduction in bacteria that maintains the gut barrier integrity. While Zhang et al.^32^ observed the beneficial effects of *Enterococcus* spp. in ameliorating microsporidian infections in silkworm microbiota, a negative correlation to the health of patients with cancer, as previously reported^35^, must also be considered. Therefore, the role of *Enterococcus* in patient health and microsporidian infection cannot be fully elucidated in this study and, thus, requires further investigations.

Like many other tropical countries, Malaysia faces challenges related to parasitic infections. This research highlights the importance of including the detection of intestinal parasites in routine diagnostic tests, considering the persistent prevalence of these infections among cancer patients in Malaysia. By exploring the relationship between intestinal parasitic infections (IPIs) and gut microbiota in Malaysian cancer patients, valuable insights can be gained into the dynamic changes caused by these infections and their impact on the gut microbial community. Analysing the altered taxa in this study contributes to a better understanding of the metabolites synthesised in response to these infections. Despite the documented dysbiosis of the gut microbiota in cancer patients, as evidenced in various literature, this research demonstrates that the intestines still play a substantial role in influencing the gut microbiota composition.

In this study, we acknowledge several limitations. Firstly, due to the strict procedures and movement control orders imposed during the COVID-19 lockdown, only single stool samples were collected from patients for convenience just a few months after sample collection began. Moreover, many patients were reluctant to participate in prolonged research due to ongoing treatment and emotional distress related to their health conditions, potentially underestimating the actual prevalence of IPIs^36^. Future work should consider examining three consecutive stool samples from similar patients. This study could also not recruit sample match pairs for the healthy control due to the COVID-19 restrictions. Thus, the datasets from healthy subjects used in this study were obtained from a previously published article by our group^37^. Healthy control included were of Malaysian locality to minimize environmental variance that may influence the gut microbiota. This study also did not collect data on cancer types, stages, chemotherapy cycles, and treatment duration due to ethical approval limitations restricted access to the patient medical records. We hope future studies will consider these factors based on our baseline findings, as reported here.

Additionally, the study did not collect information on patient diets, an important factor in understanding dysbiosis in cancer patients^38^, which should be considered in future research. Furthermore, as this study is not longitudinal, rapid microbiota changes in cancer patients due to the influence of intestinal parasites could not be captured. A longitudinal study would require patient follow-up, potentially leading to a lack of cooperation. Moreover, since participants were recruited from a single hospital, potential regional variations in gut microbiota could not be assessed. Therefore, multi-centre sample collection would be necessary to validate the present findings further. Another limitation was the small number of participants per group. Previous research has shown inconsistent results in gut microbiota composition in different types of cancer, potentially due to certain cancer types weakening the patient immune system or other treatment protocols. However, to ensure sample homogenisation, the findings could not be extrapolated based on cancer types to determine the effects of IPIs on different cancer types.

Last but not least, the detection of parasites relied on microscopy examination. We recognise that in cases where microscopy is not adequate due to the morphological similarities of the parasites, advanced molecular and immunological techniques are often required. However, the choice of method depends on factors such as availability, cost, and the specific requirements of the diagnostic setting. While our study only utilised microscopy, it remains a gold-standard diagnostic method for parasitology. For example, the concentration technique is known for its high sensitivity. Additionally, we employed the permanent staining procedure, a common practice in diagnostic laboratories for intestinal parasite infections. Nonetheless, it is important to consider other advanced screening techniques to obtain a more accurate result, which should be explored in future studies.

In conclusion, this study provides a preliminary understanding of the impact of intestinal parasites on the gut microbiota of cancer patients in a Malaysian hospital. Despite the limited samples, the study observed significant differences in gut microbiota composition in patients infected with *Cryptosporidium* spp., *Entamoeba* spp., and microsporidia compared to healthy controls. This study observed that microsporidia spp. may promote the enrichment of potentially opportunistic pathogenic bacteria from the genus *Enterococcus* in cancer patients. Conversely, while *Cryptosporidium* spp. and *Entamoeba* spp., are associated with an enrichment in metabolite-producing bacteria, microsporidia infections appear to diminish these bacteria. This nuanced understanding may be important for future research and developing therapies such as probiotics, which are tailored to the needs of cancer patients. Moreover, this study underscores the importance of acknowledging intestinal parasites as significant contributors to alterations in the microbiota in future research. Further research should aim to understand better the relationship between gut microbiota and intestinal parasites in cancer patients using a more comprehensive dataset.

## Methods

### Ethical approval

This study was approved by the Medical Ethics Committee, University of Malaya Medical Centre (UMMC), [MREC ID NO: 2019528-7454]. Before the study commencement, participants received an oral briefing from the investigator regarding the objectives and methodology of the study. All procedures adhered to relevant guidelines and regulations sanctioned by the Ethics Committee. Participants were assured that the methods employed carried no inherent risks and that their identities would remain confidential. It was explicitly stated that participation was voluntary, allowing them to withdraw without explanation. For participants aged 16 and above, informed consent was acquired through written signatures or verbal confirmation, followed by a thumbprint for those who were illiterate. In cases of participants under 16 years, informed consent was obtained from a parent or legal guardian through signed documentation or a thumbprint.

### Sample design

Over 12 months, a convenient sampling method was utilised to gather 134 stool samples from cancer patients aged one and above at the Oncology Unit, UMMC. The inclusion criteria required participants to be diagnosed with cancer, either newly diagnosed or receiving active treatment, to have refrained from taking antibiotics within a month before sample collection, and to have written consent. Demographic information such as age, gender, personal identification, diagnosis, and date of cancer therapy were obtained from their hospital records. Patients not meeting the selection criteria were excluded. To investigate the connection between IPIs and gut microbiota, the gut microbiota of stool samples that tested positive for parasites were compared with negative stool samples, using healthy controls as a baseline. The study included 33 microscopically positive stool samples and 20 negative samples. A sample was considered positive if at least one parasite was observed microscopically. Due to the COVID-19 restriction, sample match pairs of healthy controls could not be recruited. Thus, the datasets from healthy subjects were obtained from a previously published article by our team^37^. The study inclusion criteria for the control group (i.e., baseline) include healthy individuals from Malaysia with no history of parasitic diseases or cancer.

### Sample collection, microscopic examination, and DNA extraction

Single stool samples were collected in a screw-capped container and divided into fresh and fixed portions in 2.5% potassium dichromate (1:1 dilution). The freshly obtained samples were immediately stored at − 80 °C for microbiome analysis, while the preserved samples were kept at 4 °C for microscopic examination. Due to the COVID-19 lockdown, movement was restricted, and laboratory access was limited. This led to the prompt preservation of all collected stool samples in potassium dichromate and refrigeration before examination, rendering them unsuitable for culture techniques. Consequently, we selected the most appropriate method based on the study objectives and the available resources given the prevailing circumstances. As a result, more sensitive techniques, particularly for hookworm and *Strongyloides*, such as the Harada Mori culture, could not be performed during the study period as it required a fresh sample and daily monitoring.

The stool samples underwent processing using direct smear and formalin-ether concentration technique for ova, trophozoites, or cysts. Permanent staining methods such as modified Ziehl–Neelsen and Gram-chromotrope Kinyoun (GCK) staining were then used to detect intestinal protozoa. Following the manufacturer’s protocol, DNA was extracted from fresh stool samples using the FavorPrep™ Stool DNA Isolation Mini kit (Favorgen®, Taiwan). The concentration and quality of the extracted DNA were measured using NanoDrop Spectrophotometers (NanoDrop Technologies, USA). Finally, the extracted DNA was stored at − 20 °C until further use.

### Amplification and sequencing of variable 3 to 4 (V4) region of 16S ribosomal RNA (rRNA) genes

As previously described, the V3–V4 rRNA paired-end sequencing was conducted on genomic DNA extracted using gene-specific sequences from Klindworth et al.^39^. The forward primers (5ʹ-TCG TCG GCA GCG TCA GAT GTG TAT AAG AGA CAC CTA CGG GNG GCW GCA G-3ʹ) and reverse primers (5ʹ-GTC TCG TGG GCT CGG AGA TGT GTA TAA GAG ACA GGA CTA CHV GGG TAT CTA ATC C-3ʹ) containing Illumina adapter overhang nucleotide sequences (forward overhang: (5ʹ TCG TCG GCA GCG TCA GAT GTG TAT AAG AGA CAG-[locus specific sequence]) and reverse overhang: (5ʹ GTC TCG TGG GCT CGG AGA TGT GTA TAA GAG ACA G-[locus specific sequence])) were used to amplify 460 bp fragments of V3-V4 regions of the bacterial 16S rRNA gene.

A PCR reaction was performed in triplicate in a 25 μL reaction mixture containing 12.5 μL of 2× KAPA HiFi HotStart Ready Mix, 5 μL of each forward and reverse primer (1 μM), and 5 ng of template DNA PCR amplifications consisting of 3 min of initial denaturation at 95 °C, followed by 25 cycles of denaturation at 95 °C for 30 s, annealing at 55 °C for 30 s, extension at 72 °C for 30 s and final elongation at 72 °C for 5 min. According to the manufacturer’s instructions, PCR products were purified with AMPure XP beads (Beckman Coulter, U.S.). The extracted DNA quantity and quality were analysed by fluorometer with dsDNA binding dyes using Agilent DNA 1000 Kit (Agilent, Germany). Sample libraries were constructed and pooled in equimolar and paired-end sequences (2 × 250 bp) on an Illumina Miseq platform.

### 16S rRNA gene sequences processing

The raw FASTQ. files containing paired-end sequences were imported into the Quantitative Insights into Microbial Ecology 2 (QIIME2) software package version 2021.4^40^ for demultiplexing, trimming, and filtering low-quality bases. Initially, the raw sequence data were demultiplexed to remove the barcode sequence. The demultiplexed Illumina paired ends were assembled, and paired-end reads overlapping by more than 10 bp were trimmed for Illumina adapters and primers. Readings that could not be assembled and contained 2 nucleotide mismatches in primer matching were discarded. The trimmed paired-end reads were joined using a q2-vsearch plugin^41^ and filtered based on the quality score. Joined reads were truncated at any site, receiving an average quality score of < 20, and the truncated reads shorter than 50 bp containing ambiguous characters were removed.

The joined reads were denoised using Deblur (q2-deblur plugin)^42^ to filter out noisy sequences, remove chimeric sequences, remove singletons, and dereplicate the sequences to produce feature data and a table known as Amplicon Sequence Variant (ASV). The ASV feature data were rarefied to 5,191 reads per sample for further downstream analyses (q2-feature table rarefy plugin)^43^. Rarefied ASVs were aligned by mafft alignment, and FastTree was applied to generate a phylogenetic tree (q2-phylogeny plugin)^44^.

### 16S rRNA gene sequence diversity analysis

Samples were assessed for alpha diversity (variation in community composition between samples) and beta diversity (microbial diversity within samples) using metrics available in q2-diversity plugins. A Kruskal–Wallis pair-wise statistic was employed for cross-sectional analysis to assess the significance of alpha diversity and differences between groups. A p-value of less than 0.05 was considered significant^45^. Meanwhile, beta diversity was measured using weighted and unweighted UniFrac distances. Additional tests were conducted using Bray–Curtis (quantitative) and Jaccard (qualitative) distances to quantify microbial dissimilarity between samples. Subsequently, principal coordinate analysis (PCoA)^46^ was conducted on the distance matrices to identify environments that could influence the grouping of similar communities. The resulting PCoA plots were visualised using the “ggplot2” package in R. To test for significance in group distances, PERMANOVA tests with 999 permutations were utilised^47^.

Furthermore, the ASVs were taxonomically classified using a q2-feature classifier against the pre-trained Greengenes 13_8 core database 99% OTUs reference sequences, trimmed for the 16S rRNA V3-V4 regions. Taxonomic analyses were performed at the phyla and genus levels. The statistical significance of differentially abundant phyla and genera between groups was assessed using the Kruskal–Wallis test. The resulting p-values were corrected for false discovery rates using the p.adjust function with the Benjamin–Hochberg method implemented in R. All results were visualised using the “ggplot2” package in R.

### Identification of confounding variables

Several host phenotypic and environmental variables, such as age, gender and diet, have been linked to differences in gut microbiota composition. In this study, no information on the patient diet was obtained. Furthermore, given the differences in the characteristics between patient cohorts, preliminary diversity analyses were performed to identify the confounding variable and minimise bias in our analysis. In this study, significant differences were observed between symptomatic and asymptomatic samples. Thus, symptomatic samples were excluded from this study. No significant differences in microbial richness and composition were observed in other pairs of groups regardless of gender, race, cancer group and the status of cancer treatment.

### LDA effect size (LefSe) analysis

The LefSe analysis, conducted using the Galaxy online interface (http://huttenhower.sph.harvard.edu/galaxy/), aimed to identify the key bacterial taxa showing differential abundance between microbiome pairs. The comparison classes were based on the types of parasite infections, comparing cohorts of infected and non-infected patients. Initially, LefSe identified statistically different features among the biological classes. Subsequently, a non-parametric factorial Kruskal–Wallis (KW) rank-sum test was performed, and a linear discriminant analysis model was used to estimate the effect sizes of the identified features, determining whether they are consistent with the expected behaviour of the different biological classes^48^.

### Statistical analysis

Data were analysed using Excel (Microsoft Corporation, US) and SPSS software (Statistical Package for the Social Sciences, version 25.0, SPSS Inc Chicago, III, USA). Demographic data, including age, gender, personal identification, diagnosis, and date of cancer therapy, were treated as categorical variables. Categorical variables were presented as frequency (per cent) and 95% confidence intervals (95% CI). When appropriate, a Chi-square or Fisher’s exact test was conducted to identify any differences among the variables. A p-value of less than 0.05 was considered significant.

### Supplementary Information

Supplementary Table S1.

## Data Availability

The raw sequence data reported in this study have been deposited in the Genome Sequence Archive^49^ in the National Genomics Data Center^50^, China National Center for Bioinformation/Beijing Institute of Genomics, Chinese Academy of Sciences (GSA-Human: HRA006733) that are publicly accessible at https://ngdc.cncb.ac.cn/gsa-human.
