# Supplementary material for: Study on intestinal parasitic infections and gut microbiota in cancer patients at a tertiary teaching hospital in Malaysia
Source: Sci Rep. 2024 Jun 13;14:13650. doi: 10.1038/s41598-024-59969-6 (PMC11176305; doi:10.1038/s41598-024-59969-6)
Supplement: Supplementary file 1 — Supplementary Table S1. [file 41598_2024_59969_MOESM1_ESM.docx]

**Supplementary Table S1. Demographic characteristics of cancer patients involved in the study (N = 134)**

| **Characteristic** | **n** |
| --- | --- |
| **Age**  Range  Median (IQR)  Mean (SD) | 1 – 96  25 (8 – 51)  30.8 (25.9) |
| **Age groups**  1 – 20  21 – 40  41 – 60  61 – 80  81 – 100 | 65 (48.5%)  25 (18.7%)  25 (18.7%)  12 (9%)  7(5%) |
| **Gender**  Male  Female | 75 (56%)  59 (44%) |
| **Race**  Malay  Chinese  Indian  Others | 60 (44.8%)  52 (38.8%)  20 (14.9%)  2 (1.5%) |
| **Symptoms**  Asymptomatic  Diarrhoea  More than one  Nausea | 64 (47.8%)  55 (41%)  12 (9%)  3 (2.2%) |
| **Cancer Types**  **Solid tumour**  Uncharacterised solid tumour  Medulloblastoma  Ewing Sarcoma  Osteosarcoma  Germinoma  Angiofibroma  Breast cancer  LCH  Ovarian cancer | 54 (40.3%)  6 (4.5%)  3 (2.2%)  2 (1.5%)  2 (1.5%)  1 (0.7%)  1 (0.7%)  1 (0.7%)  1 (0.7%) |
| **Haematological malignancies**  Uncharacterised leukaemia  ALL  AML  Lymphoma  JMML | 27 (20.1%)  27 (20.1%)  6 (4.5%)  2 (1.5%)  1 (0.7%) |
| **Cancer groups**  Solid tumour  Haematological malignancies | 70 (52.2%)  64 (47.8%) |
| **Cancer Treatment**  Yes  No | 119 (88.8%)  15 (11.2%) |
| **Down Syndrome**  No  Yes | 129 (96.3%)  5 (3.7%) |

N = Total number of samples; n = number of samples examined; IQR = Interquartile range; SD = Standard deviation; ALL = acute lymphoblastic leukaemia; AML = acute myeloid leukaemia; JMML = juvenile myelomonocytic leukaemia; LCH = Langerhans cell histiocytosis
